# Inhibition of CREB‐mediated ZO‐1 and activation of NF‐κB‐induced IL‐6 by colonic epithelial MCT4 destroys intestinal barrier function

**DOI:** 10.1111/cpr.12673

**Published:** 2019-08-16

**Authors:** Shunxian Zhang, Wanfu Xu, Hongli Wang, Meiwan Cao, Musheng Li, Junhong Zhao, Yan Hu, Yaodong Wang, Songyu Li, Yuanwen Xie, Guanhua Chen, Ruitao Liu, Yang Cheng, Zhaohui Xu, Kejian Zou, Sitang Gong, Lanlan Geng

**Affiliations:** ^1^ Guangzhou Institute of Pediatrics, Guangzhou Women and Children’s Medical Center Guangzhou Medical University Guangzhou China; ^2^ Department of Gastroenterology, Guangzhou Women and Children’s Medical Center Guangzhou Medical University Guangzhou China; ^3^ Department of Anesthesiology Hainan General Hospital Haikou China; ^4^ Department of Gastroenterology Kunshan affiliated Hospital of Nanjing University of Chinese Medicine Kunshan China; ^5^ Department of Clinical Laboratory Qionghai Hospital of Traditional Chinese Medicine Qionghai China; ^6^ Department of Anorectal Qionghai Hospital of Traditional Chinese Medicine Qionghai China; ^7^ Department of General Surgery Hainan General Hospital Haikou China

**Keywords:** CREB, IL‐6, inflammatory bowel disease, monocarboxylate transporter 4, nuclear factor‐κB, ZO‐1

## Abstract

**Objective:**

Inflammatory bowel disease (IBD) is a disorder intestinal inflammation and impaired barrier function, associated with increased epithelial expression of monocarboxylate transporter 4 (MCT4). However, the specific non‐metabolic function and clinical relevance of MCT4 in IBD remain to be fully elucidated.

**Methods:**

Lentivirus‐mediated overexpression of MCT4 was used to assess the role of MCT4 in transcriptionally regulating ZO‐1 and IL‐6 expression by luciferase assays, WB and ChIP. IP was used to analyse the effect of MCT4 on the interaction NF‐κB‐CBP or CREB‐CBP, and these MCT4‐mediated effects were confirmed in vivo assay.

**Results:**

We showed that ectopic expression of MCT4 inhibited ZO‐1 expression, while increased pro‐inflammatory factors expression, leading to destroy intestinal epithelial barrier function in vitro and in vivo. Mechanistically, MCT4 contributed NF‐κB p65 nuclear translocation and increased the binding of NF‐κB p65 to the promoter of IL‐6, which is attributed to MCT4 enhanced NF‐κB‐CBP interaction and dissolved CREB‐CBP complex, resulting in reduction of CREB activity and CREB‐mediated ZO‐1 expression. In addition, treatment of experimental colitis with MCT4 inhibitor α‐cyano‐4‐hydroxycinnamate (CHC) ameliorated mucosal intestinal barrier function, which was due to attenuation of pro‐inflammation factors expression and enhancement of ZO‐1 expression.

**Conclusion:**

These findings suggested a novel role of MCT4 in controlling development of IBD and provided evidence for potential targets of IBD.

## 1 INTRODUCTION

Despite tremendous efforts have been made to improve the effectiveness of treatment, inflammatory bowel disease (IBD), including ulcerative colitis (UC) and Crohn's disease (CD), remains the leading cause of cancer‐related healthy people in the world.^1^, ^2^ Growing evidences showed that IBD is a disorder of dysregulation by inflammation accompanied by impaired intestinal barrier function,^3^, ^4^, ^5^ which is an vital event in treatment of IBD. For instance, pro‐inflammatory cytokine IL‐6 and TNF‐α increased intestinal epithelial cell shedding and apoptosis, potentially challenging the barrier between the gastrointestinal lumen and internal tissues.^6^ In addition, disruption of tight junction is regarded as one of the earliest hallmarks of epithelial injury, leading to the loss of cell polarity and tissue disorganization.^7^ Thus, deciphering the key molecules that enable anti‐inflammatory and protection of intestinal barrier function are urgently needed to improve therapeutic outcomes.

A key intracellular signalling pathway that governs intestinal barrier function is MAPK signalling. The major downstream transcription factors of the MAPK pathway are the cAMP‐response element binding protein (CREB) and nuclear factor‐κB (NF‐κB).^8^, ^9^ Phosphorylation of CREB at ser133, but not Ser142, directly or indirectly activated by ERK1/2, p38MAPK and other stimuli,^10^, ^11^, ^12^ increases its transcriptional activity and target genes expression, such as muc2^13^ and tight junction protein 1 (ZO‐1).^14^, ^15^ Phosphorylation at this residue promotes CREB binding to transcriptional co‐activator CBP, which lead to displacement of NF‐κB from the same interaction domain on CBP.^16^, ^17^ Formation of CREB‐CBP complex promotes the expression of anti‐inflammatory cytokines(eg, IL‐4, IL‐10 and IL‐13),^18^ thereby suppressing expression of pro‐inflammatory cytokines(eg, IL‐6 and TNF‐α) activated by the NF‐κB‐CBP complex,^12^, ^19^ suggesting the biological action is maintained in part by the activity of CREB and NF‐κB. Nevertheless, the upstream and role of both in intestinal epithelial barrier function have not yet been fully illustrated.

Recent study have showed that proliferative cells at the base of the intestinal crypt are characterized by a glycolytic metabolic phenotype,^20^ whereas differentiated cells have an oxidative phosphorylation phenotype, which is in line with the findings showed by sun et.al^21^ and Wang et.al,^22^ implying that nutritional states are closely associated with alteration of intestinal barrier function. Interesting, our previous study has revealed monocarboxylate transporter 4 (MCT4) expression is significantly increased in intestinal mucosal tissue of IBD patients, which is correlated with intestinal mucosal inflammation,^23^ and MCT4 is involved in establishment and maintenance of epithelial polarity and lactate transporters.^24^, ^25^ However, the effect of MCT4 that has a global influence on IBD has not yet been illustrated. In this study, we further demonstrated that MCT4 destroyed intestinal barrier function by reduction of ZO‐1 expression and promotion of pro‐inflammatory IL‐6 expression in vivo and in vitro. Mechanically, ectopic expression of MCT4 significantly inhibited phosphorylation of CREB(Ser133), leading to reduce ZO‐1 expression, while promoted phosphorylation of Ser536 on p65, resulting in enhancement of IL‐6 expression. Most importantly, endogenous CBP interacted with CREB, and this interaction was dramatically disrupted by ectopic expression of MCT4, which in turn promotes CBP‐NF‐κB complex. What's more, α‐cyano‐4‐hydroxycinnamate (CHC), an inhibitor of MCT4, alleviated dextran sulphate sodium (DSS)‐induced colitis in vivo. Collectively, these findings provide the mechanism by which MCT4 regulated IBD and support MCT4 inhibitor used as an effective therapeutic approach to improve IBD.

## 2 MATERIALS AND METHODS

### 2.1 Reagents and antibodies

CaCO_2_ and HT‐29 cells were obtained from American Type Cell Collection (Manassas, VA). Cell culture medium and foetal bovine serum were purchased from Life Technologies, and α‐cyano‐4‐hydroxycinnamate (C2020) was supplied by Sigma. Antibody against MCT4 (sc‐50329) and CBP (sc‐7300 X) were from Santa Cruz Biotechnology. TJP1 (A0659), IL‐6 (A2447), IL‐10 (A2171), TNF‐α (A0277), phospho‐CREB (Ser133) (AP0333), CREB (A10826), E‐cadherin (A3044), Occludin (A2601), TJP2 (A0594), Claudin1 (A2196), Claudin2 (A11843), Claudin5 (A10207), Claudin7 (A2035), Claudin8 (A8174), Claudin14 (A2948), JAMA (A1241), Histone 3 (A2348) and alpha‐tubulin (AC013) were purchased from Abclonal. Phospho‐p65 (Ser536) (No.3033) and p65 (No.8242) were purchased from Cell Signaling Technology. Other reagents used in this study were purchased from Sigma.

### 2.2 Plasmid and siRNA

Plasmid and siRNA‐targeted NF‐κB p65 were purchased from GenePharma.

### 2.3 Cell culture and transfection

CaCO_2_ and HT‐29 cell line was cultured in DMEM supplemented with 10% foetal bovine serum and maintained in a humidified incubator at 37°C and 5% CO_2_. For transfection, plasmids or siRNA was transfected into cells with hilymax (H357) and lipofectamine3000 (L‐3000), respectively, following the manufacturer's instructions.

### 2.4 Lentivirus‐mediated overexpression of MCT4

The lentivirus vectors Lv‐CTL and Lv‐MCT4 were purchased from Genepharma, and puromycin was purchased from Sigma and used to select for stably transfected cells. MCT4 expression at protein level was verified by Western blotting.

### 2.5 RNA Extraction and quantitative real‐time PCR

Total RNA was extracted using Trizol (life technologies), converted to cDNA using the All‐in‐One™ First‐Strand cDNA Synthesis Kit (Genecopoeia™, FulenGen) and amplified by PCR using the All‐in‐One™ qPCR Mix (Genecopoeia™, FulenGen) according to the manufacturer's instructions. Primer sequences for *Claudins* were from Sangon Biotech, and other primers for indicated genes used in this study were listed in Table 1.

**Table 1:** primer for indicated genes in real‐time PCR assay

Gene	Forward	Reverse
ZO‐1	TGTGAGTCCTTCAGCTGTGGAA	GGAACTCAACACACCATTG
IL‐1β	GTAGCCCACGTCGTAGCAAA	CCCTTCTCCAGCTGGGAGAC
IL‐6	GGAACTCAACACACCATTG	GTCAGGTCGGACTCCCGAGAA
IL‐8	GCAGCTCTGTGTGAAGGTGCAGTTT	CTCAGCCCTCTTCAAAAACTTCTCC
TNF‐α	CAGAGGGAAGAGTTCCCCAG	CCTTGGTCTGGTAGGAGACG
COX‐2	TGCATTCTTTGCCCAGCACT	AAAGGCGCAGTTTACGCTGT
IL‐10	ACAGGGAAGAAATCGATGACA	TGGGGGAGAACCTGAAGAC
MCT4	GAGTTTGGGATCGGCTACAG	CGGTTCACGCACACACTG
TJP2	GGGAAGGTCGCTGCTATTGT	CTCTCGCTGTAGCCACTCC
JAM	TGTTTCAGTTCTGTGTCATGGT	TGCAGACAAGGTGTTTTCCAG
Occludin	ACAAGCGGTTTTATCCAGAGTC	GTCATCCACAGGCGAAGTTAAT
E‐cadherin	TGCCCAGAAAATGAAAAAGG	GTGTATGTGGCAATGCGTTC
UBC	ATTTGGGTCGCGGTTCTTG	TGCCTTGACATTCTCGATGGT

### 2.6 Immunoprecipitation and immunoblotting

As described in our previous study, cells were lysed in ice‐cold buffer composed of 50 mmol/L Tris‐HCl (pH 7.4), 150 mmol/L NaCl, 0.1% Nonidet (NP‐40) and protease inhibitors. Lysates were incubated with primary CBP antibody overnight at 4°C, and the immune complexes were pulled down with fresh protein A/G. The bound proteins were eluted in denaturing SDS sample buffer and analysed by Western blotting. Protein concentrations were determined using Pierce BCA Protein Assay Kit (Thermo Fisher Scientifics), prepared in 2xSDS sample buffer, subjected from SDS‐PAGE and transferred to a 0.22‐µm nitrocellulose transfer membrane. The membrane was blocked with 5% (w/v) milk in PBS/0.05% (v/v) Tween‐20 and incubated with the indicated antibody overnight at 4°C followed by incubation with a horseradish peroxidase secondary antibody (Jackson ImmunoResearch) for 1 hour at room temperature. Proteins were detected using an enhanced chemiluminescence (Perkin Elmer).

### 2.7 Enzyme‐linked immunosorbent assay

Cytokines, including TNF‐α, IL‐6, IL‐1β, IL‐8, IL‐10 and COX2 in culture supernatants and TNF‐α, IL‐6 and IL‐1β in serum of mice, were quantitated for the indicated group by sandwich ELISA according to the manufacturer's instructions (Elabscience Biotechnology).

### 2.8 Luciferase reporter assay

Cells were co‐transfected with the indicated promoter reporter plasmid (ZO‐1‐luc or IL‐6‐Luc) and internal control plasmid (pGL4.74[hRluc/TK], from Promega). Twenty‐four hours after transfection, Firefly and Renilla luciferase value in cells were measured using the Dual‐Luciferase Reporter Assay System (Promega).

### 2.9 Chromatin immunoprecipitation

ChIP assays were performed using SmapleChIP(R) Plus Kit (Magnetic Bead) (Cell Signal Technology, 9005) as described in our pervious study. Lysates were incubated with primary antibodies or negative control anti‐IgG. The precipitated DNAs were analysed and quantified by using real‐time PCR analysis. Primers to amplify the proximal region of the ZO‐1 promoter containing a CREB‐binding site were 5ʹ‐CTTGAGGTCTAATGTGGGGTG‐3ʹ and 5ʹ‐CATGGCTTTCATCTCCGAG‐3ʹ^14^. The primers for IL‐6 promoter containing a p65 binding site were 5ʹ‐CGGTGAAGAATGGATGACCT‐3ʹ and 5ʹ‐AAACCAGACCCTTGCACAAC‐3ʹ.

### 2.10 DSS‐induced colitis and Intestinal Permeability Assays

Colitis mice were induced by 2% (w/v) dextran sulphate sodium (DSS, Millipore Corporation) in drinking water for 5 days, followed by CHC or vehicle treatment for a period of 14 days. The body weight changes and overall survival were recorded daily. On day 20, fluorescent conjugated dextran (10 kDa with Cy7) was gavaged into mice, and then, 1 hour later animals were studied using multispectral fluorescent capture followed by digital X‐ray imaging.

### 2.11 In vitro barrier function assessment

Twelve‐well plate Millicells (0.4 μm, Millipore Corporation) were used for transepithelial electrical resistance (TEER) assays as described previously. Briefly, 0.5 mL CaCO_2_ cells at the density of 4 × 10^5^ cells/mL were seeded in the apical chamber that bathed in the basal chamber with 1.0 mL DMEM complete medium for 21 days. Voltage was measured daily using EVOM (WPI), which was multiplied by the area of filter (1.12 cm^2^) to obtain the TEER in Ohm cm^2^. DMEM complete medium in apical and basal chamber was refreshed every day. The permeability of FITC‐dextran (Sigma) across the CaCO_2_ cell monolayer was measured as previously described with modifications. At 21 days, 1.0 mg/mL FITC‐dextran was added on the apical side of monolayers after washed twice with PBS. One millilitre cells in the basal chamber were taken at indicated point, and 1.0 mL pre‐warmed fresh medium was added after each sampling to replenish basal medium. The fluorescence emission at 520 nm was measured with excitation at 490 nm using Synergy H1 microplate reader.

### 2.12 Immunohistochemistry (IHC) and immunofluorescence(IF)

Immunohistochemistry and immunofluorescence were performed as described in our previous work.^26^, ^27^ The sections were deparaffinized, rehydrated, blocked with goat serum and incubated with indicated antibody at 4°C overnight, the bound antibodies were then visualized using diaminobenzidine as a chromogen, and the slides were counterstained with haematoxylin. The area of positive staining was measured in six different images taken at 400× magnification on each slide and quantified using Image‐Pro Plus 6.0 software (Media Cybernetics). While for immunofluorescence staining, after incubation with the primary antibodies, the section was washed with PBS and incubated with appropriate fluorescent secondary antibodies. Sections were mounted using DAPI and imaged by fluorescent microscopy.

### 2.13 Statistical analysis

All analysis was conducted using GraphPad Prism V software. A *P* value < .05 was considered statistical significant. Statistical differences among groups were determined by Student's *t* test, one sample *t* tests, one‐way ANOVA or two‐way ANOVA were used to determine the significance.

## 3 RESULTS

### 3.1 MCT4 is significantly increased in the colonic epithelium of IBD patients and DSS‐induced colitis

**Figure 1:**
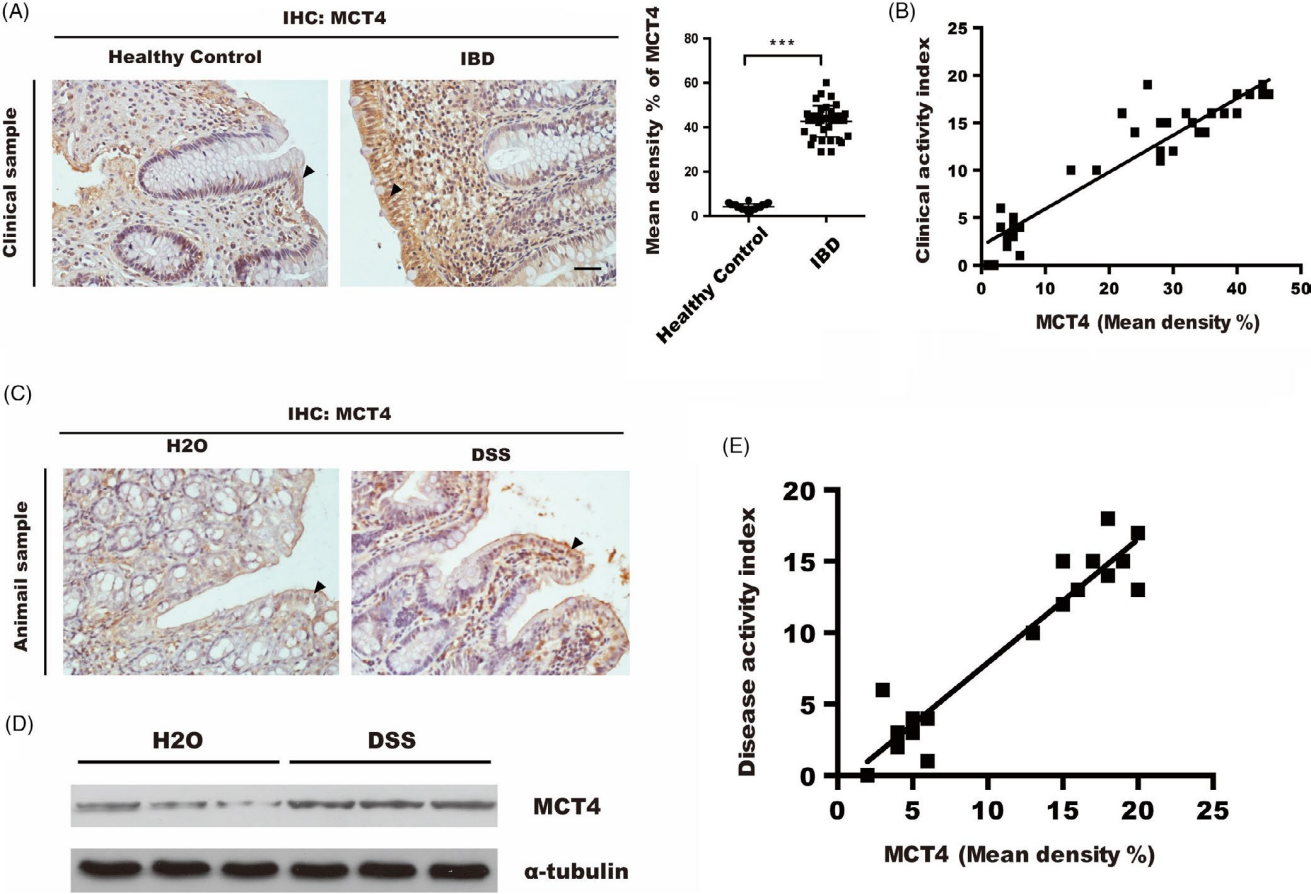
MCT4 is increased in human UC and DSS‐induced experimental colitis. A, Representative sections were prepared from colonic mucosa of healthy control and inflammatory bowel disease (IBD) patients and stained for MCT4 expression, ***P < .001, scale bar: 50 μm. B, Correlation between MCT4 in colon biopsies and clinical activity index. C‐D, MCT4 expression was analysed via IHC and immunoblotting in indicated treatment. E, Correlation between MCT4 in DSS‐induced colitis and disease activity index. Scale bar: 50 μm

In previous study, we demonstrated that a higher levels of MCT4 expression in intestinal mucosa of IBD patients were compared with healthy controls.^23^ Consistently, as shown in Figure 1A, immunohistochemical staining revealed a major source of MCT4 in colonic epithelium of IBD patients (n = 54) compared with that in healthy controls (n = 16), and the MCT4 expression was positively correlated with clinical activity index (Spearman *R* = 0.891,*P* < .001) (Figure 1B). In line with this, Western blotting and immunohistochemistry showed that MCT4 expressions were enhanced in DSS‐induced colitis mice compared with those healthy control (Figure 1C‐D), which were correlation with disease activity index (Spearman *R* = 0.909, *P* < .001) (Figure 1E). Taken together, these data indicated high expression of MCT4 in ulcerative colitis (UC) and DSS‐induced colitis mice; however, the function of MCT4 in the development of IBD remains unknown.

### 3.2 MCT4 destroyed intestinal barrier function through suppression of ZO‐1 expression

**Figure 2:**
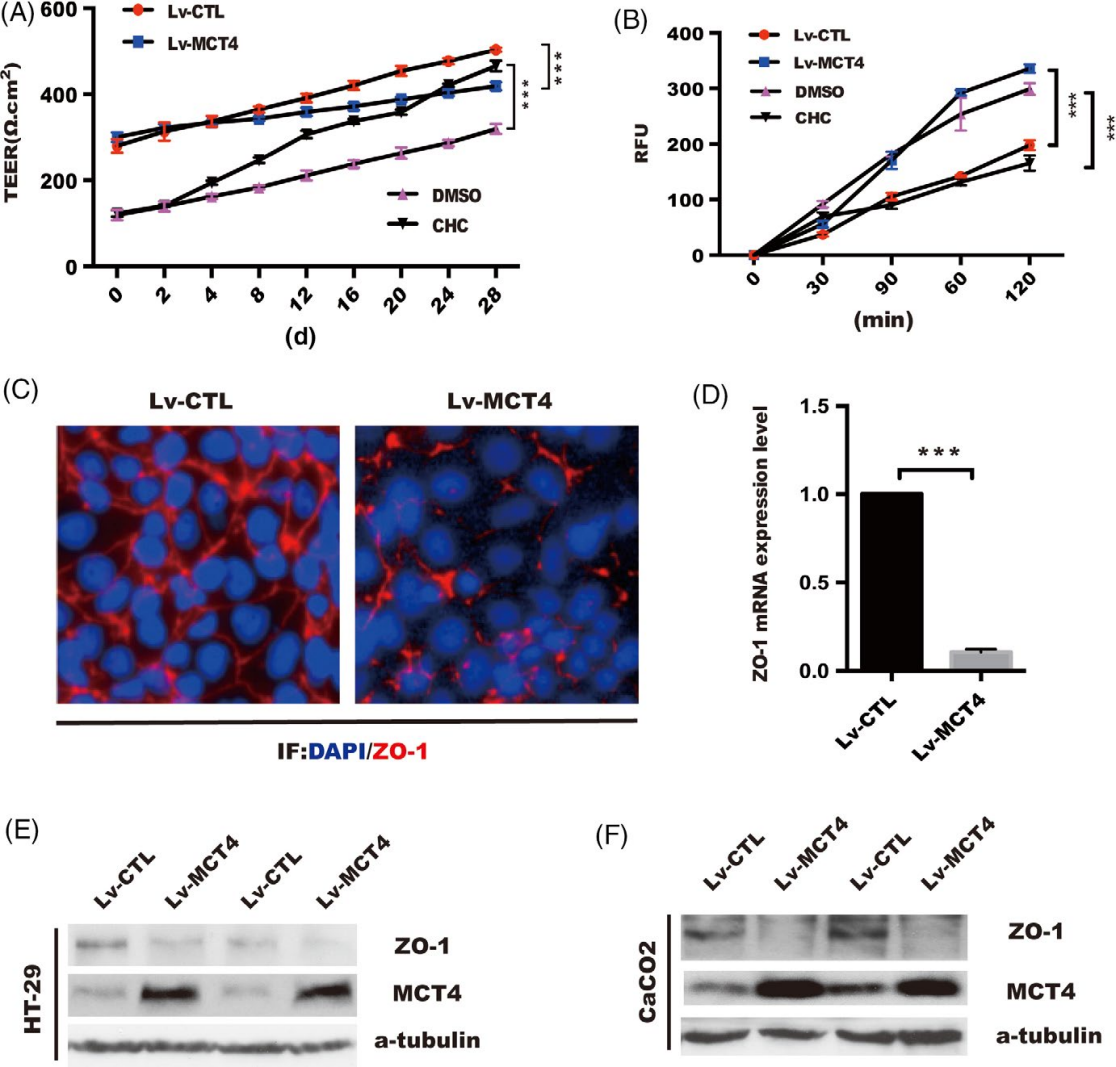
MCT4 destroys epithelial barrier function and inhibits ZO‐1 expression. A, Transepithelial electrical resistance was detected over time in CaCO_2_ cells treated as indicated. B, Cy7‐dextran paracellular intestinal epithelial permeability was performed at 28 days post incubation. C, Immunofluorescence staining of tight junction protein ZO‐1 in CaCO_2_ cells was grown to confluence in indicated group. D, ZO‐1 mRNA level was analysed in real‐time PCR analysis in CaCO_2_ cells, scale bar: 50 μm. E‐F, Western blotting was employed to detect ZO‐1 expression in stable HT‐29 and CaCO_2_ cells with MCT4 overexpression

It has been reported that dysfunction of intestinal mucosal barrier caused the invasion of luminal pathogens into the lamina propria, which further triggered the dysregulation of immune responses, leading to chronic colonic inflammation.^28^ To clarify whether upregulated MCT4 expression was involved in regulation of the epithelial barrier dysfunction, CaCO_2_ monolayers were introduced with MCT4 lentivirus (Lv‐MCT4), and the changes in barrier permeability were detected by transepithelial electrical resistance (TEER) and FITC‐dextran flux assays in transwell polyester membrane filters. As illustrated in Figure 2A, TEER value was significantly decreased in Lv‐MCT4‐treated cells compared with that in the control, whereas treated with MCT4 inhibitor (CHC) markedly increased TEER value. Consistently, FITC‐dextran flux was found to be significantly increased by overexpression of MCT4 (Figure 2B). Consistently, further results showed that MCT4 significantly inhibited ZO‐1 by immunofluorescences (Figure 2C), qPCR (Figure 2D) and Western blotting (Figure 2E‐F) after ectopic expression of MCT4 in intestinal epithelial cells; in addition, MCT4 have failed to alter TJP2 and Occludin expression at mRNA and protein level (Figure S1A), whereas slight changes in JAM, Claudins (sealing tight junction proteins claudins 1, 4, 5, 7, 8 and 14)^29^ and E‐cadherin expression were observed at protein level (Figure S1B‐C). Taken together, these data suggested that MCT4 destroyed intestinal epithelial barrier function primarily through reduction of ZO‐1.

### 3.3 MCT4 inhibited the binding of CREB to ZO‐1 promoter via reduction of phosphorylation of CREB (Ser133) and nuclear translocation

**Figure 3:**
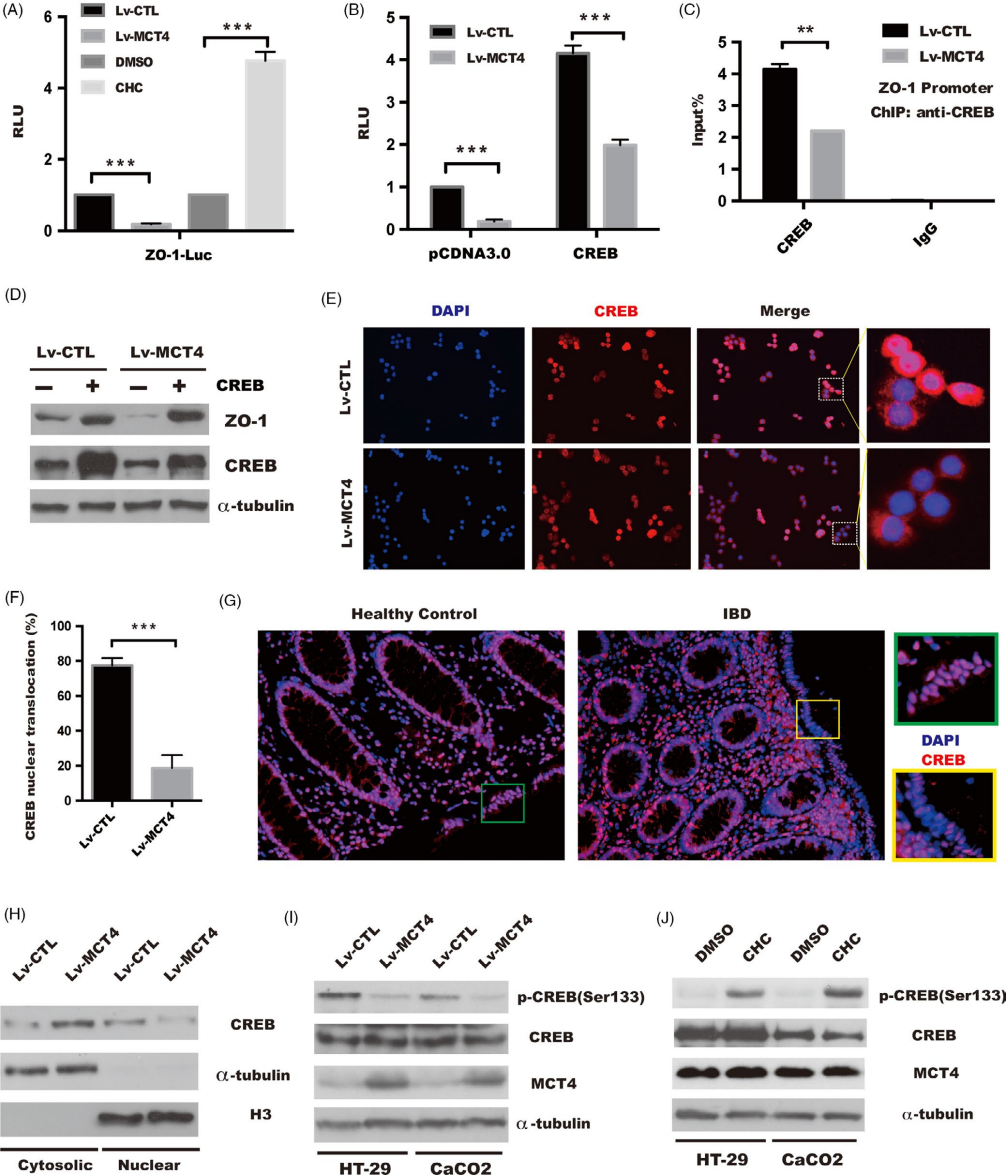
MCT4 inhibits phosphorylation of CREB(Ser133) and attenuates CREB‐mediated ZO‐1 transactivity. A, Luciferase activity in cells which was transiently transfected with the pGL4.17‐ZO‐1 promoter plasmid together with renilia plasmid in stable HT‐29 cells or in HT‐29 cell treated as indicated. The Renilla luciferase activities were used as internal controls. One‐way analysis of variance (ANOVA) and Dunnett's multiple comparison test, ***P < .001, n = 3; error bars indicate s.d. B, pGL4.17‐ZO‐1 promoter plasmid together with pCDNA 3.0 or CREB plasmid was transfected into indicated stable HT‐29 cells, and the Renilla luciferase activities were used as internal controls. Two‐way analysis of variance (ANOVA) and Dunnett's multiple comparison test, ***P < .001, n = 3; error bars indicate s.d. C, ChIP analysis of binding of CREB protein to ZO‐1 gene promoter in CaCO_2_ cells treated as indicated. Student's *t* test, **P < .05, n = 3; error bars indicated s.d. D, Western blotting was performed to analyse ZO‐1 expression in indicated CaCO_2_ cells transfected with CREB or control plasmid, α‐tubulin served as internal control. E, Immunofluorescence was employed to detect CREB nuclear translocation in indicated HT‐29 cells, scale bar: 200 μm. F, Percentage of cells that exhibited CREB nuclear translocation. Data represent the means ± s.d. of three independent experiments and were analysed by *t* test, ***P < .05. G, CREB nuclear location in intestinal tissue from patients with IBD and healthy control was performed by IF, scale bar: 100 μm. H, Levels of nuclear (Nuclear) and cytosolic (Cytosolic) CREB were determined by immunoblotting analysis. α‐tubulin and H3 were used as internal controls for the cytosolic and nuclear fractions, respectively. I‐J, Whole cell lysates were separated by SDS‐PAGE and assayed with the antibodies against the indicated proteins, inducing pCREB(Ser133), CREB and MCT4, in indicated group of HT‐29 and CaCO_2_ cells, and α‐tubulin was determined to ensure equal loading

CREB has been shown to play a central role in transactivation of ZO‐1^14^, ^15^, ^30^; therefore, we focused on MCT4‐mediated ZO‐1 expression might be attributed to downregulation of CREB signalling. As shown in Figure 3A, luciferase reporter plasmids containing a 2kb ZO‐1 promoter (ZO‐1‐Luc) were constructed and co‐transfected with pGL4.17 into indicated stable cell lines or CaCO_2_ treated with and without CHC. Overexpression of MCT4 inhibited, while treatment of CHC increased ZO‐1 promoter activity. Ectopic expression of CREB rescued the diminished ZO‐1 luminescence caused by MCT4 depletion (Figure 3B). Importantly, chromatin immunoprecipitation (ChIP) assay confirmed that the ectopic MCT4 expression reduced the binding of CREB to ZO‐1 gene promoter in HT‐29 cells (Figure 3C). Moreover, overexpression of the CREB gene using plasmid reversed the abrogation of ZO‐1 expression in CaCO_2_ cells of Lv‐MCT4 group compared with Lv‐CTL group (Figure 3D). These results indicated that MCT4 has a significant role in CREB‐mediated transactivation of ZO‐1.

Although our data suggested that MCT4 was essential for CREB‐dependent transcription of ZO‐1, the exact mechanisms remained unclear. Activation of CREB promoted its nuclear translocation to activate CREB‐dependent genes transcription,^31^ and we utilized immunofluorescence to analyse the influence of MCT4 on nuclear distribution of CREB. As shown in Figure 3E‐F, CREB nuclear translocation was decreased to 25% of that in the control cells by introduction of MCT4. Meanwhile, the level of nuclear CREB in undetectable in IBD mice mucosa was compared with that in healthy control (Figure 3G). In line with this, a subcellular fractionation analysis showed that MCT4 led to a significant increase in the amount of cytosolic CREB(Figure 3H), indicating MCT4 has an important role in regulation of CREB translocation.

Phosphorylation of CREB (Ser133) is critical for CREB translocation in response to various stimuli,^32^, ^33^, ^34^ next, we sought to further elucidate the possible mechanism underlying CREB nuclear translocation. As shown in Figure 3I; overexpression of MCT4 drastically abolished phospho‐CREB(Ser133) in HT‐29 and CaCO_2_ cells; and in contrast, inhibition of MCT4 by CHC significantly increased phosphorylation of CREB at Ser133 (Figure 3J). Taken together, these findings suggested that MCT4 repressed ZO‐1 transcription by de‐phosphorylation and reduction of CREB nuclear accumulation.

### 3.4 MCT4 contributed to upregulation of global inflammatory factors expression

**Figure 4:**
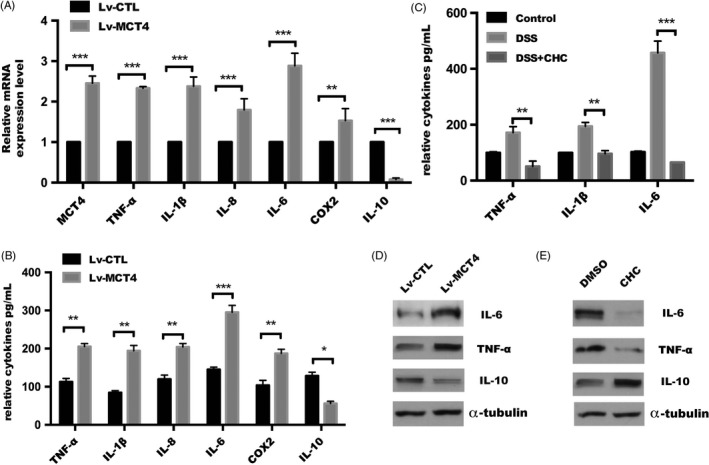
MCT4 modulates various inflammatory cytokines expression. (A) Real‐time PCR and (B) ELISA assay were performed to detect a serial of genes expression in CaCO_2_ cells. (C) Relative cytokines in serum levels assayed in mice treated as indicated. (D‐E) Western blotting was performed to detect relative cytokines expression in indicated group

MCT4 inhibitor CHC was used to block the activity of ERK and activates p38 signalling pathway and their target genes (eg, TNF‐α, IL‐6, IL‐1β and IL‐8),^35^ which prompted us to investigate whether MCT4 has a global effect on these dominant inflammatory factors vital to intestinal inflammation, and we employed a cytokines antibody array using conditioned medium controlled from stable CaCO_2_ cells. The pervious array analysis implied that ectopic expression of MCT4 caused a significant increase in most inflammatory cytokines. As shown in Figure 4A, ectopic expression of MCT4 in CaCO_2_ cells caused IL‐1β, TNF‐α and IL‐8, especially IL‐6, was increased to approximately 2‐fold of the Lv‐CTL control, which is consistent with Tan et al report,^36^ while IL‐10 was dramatically decreased to almost undetectable levels. Moreover, ELISA assay showed that MCT4 significantly upregulated IL‐6, IL‐8, IL‐1β and TNF‐α secretion, while attenuated IL‐10 level in supernatant in both HT‐29 and CaCO_2_ cells (Figure 4B). Consistent with increased access of these pro‐inflammatory factors, serum levels of IL‐1β, TNF‐α and IL‐6 were elevated in DSS‐treated mice, which were drastically reduced in mice colitis model following CHC treatment (Figure 4C). In addition, Western blotting confirmed that overexpression of MCT4 in CaCO_2_ cells strongly enhanced these pro‐inflammatory factors expression and sharply reduced IL‐10 expression, while opposite effect was obtained by CHC treatment (Figure 4D‐E). Collectively, these data suggested that MCT4 promoted the expression of pro‐inflammatory cytokines (IL‐6, IL‐8, IL‐1β and TNF‐α) and inhibited anti‐inflammatory IL‐10 levels in IECs.

### 3.5 MCT4 enhanced NF‐κB binding to IL‐6 promoter by promotion of phosphorylation and nuclear translocation of NF‐κB p65

**Figure 5:**
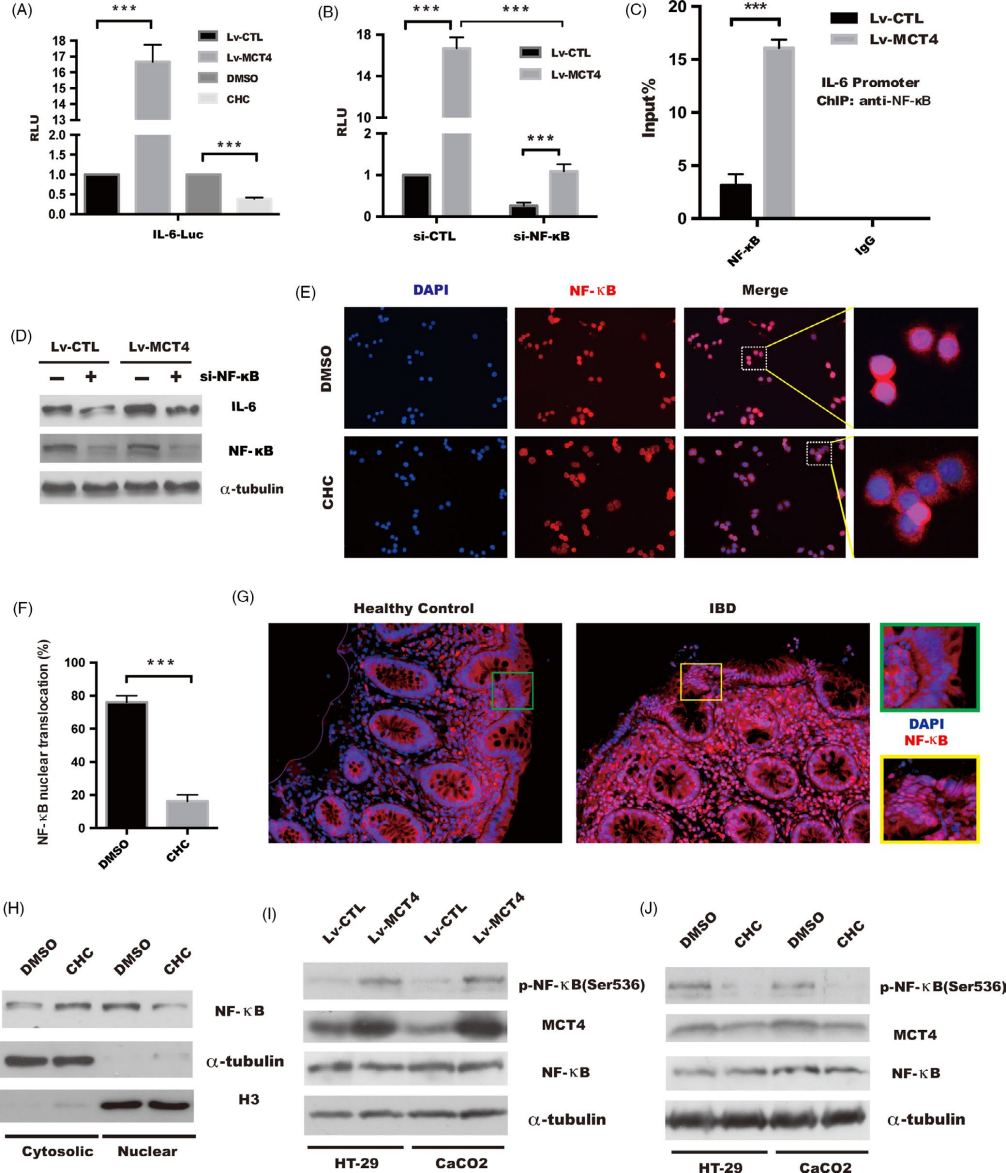
Increased phosphorylation of NF‐κB(Ser536) and NF‐κB‐induced IL‐6 transactivity in response to MCT4 overexpression. A, Luciferase activity was performed by transiently transfecting with the pGL4.17‐IL‐6 promoter plasmid together with renilia plasmid in stable CaCO_2_ cells or in CaCO_2_ cells treated as indicated, respectively. The Renilla luciferase activities were used as internal controls. One‐way analysis of variance (ANOVA) and Dunnett's multiple comparison test, ***P < .001, n = 3; error bars indicate s.d. B, pGL4.17‐IL‐6 promoter plasmid together with si‐CTL or si‐NF‐κB was transfected into indicated stable HT‐29 cells, and the Renilla luciferase activities were used as internal controls. Two‐way analysis of variance (ANOVA) and Dunnett's multiple comparison test, ***P < .001, n = 3; error bars indicate s.d. C, ChIP analysis of binding of NF‐κB protein to IL‐6 gene promoter in stable CaCO_2_ cells. Student's *t* test, ***P < .001, n = 3; error bars indicated s.d. D, Immunoblotting was performed to analyse IL‐6 expression in indicated stable CaCO_2_ cells transfected with si‐CTL or si‐NF‐κB, respectively, and α‐tubulin was used as internal control. E, Immunofluorescence of NF‐κB location in indicated HT‐29 cells treated by DMSO or CHC 1 hour, scale bar: 200 μm. F, Percentage of cells that exhibited NF‐κB nuclear translocation. Data represent the means ± s.d. of three independent experiments and were analysed by *t* test, ***P < .001. G, Immunofluorescence of NF‐κB nuclear location in intestinal tissue from patient with IBD and healthy control, scale bar: 100 μm. H, Analysis of subcellular fractionation of NF‐κB by immunoblotting analysis in CaCO_2_ cell treated with DMSO or CHC, respectively. α‐tubulin and H3 served as internal controls for the cytosolic and nuclear fractions, respectively. I‐J, Whole cell lysates were subjected to SDS‐PAGE and assayed with the antibodies against the indicated proteins in indicated group of HT‐29 and CaCO_2_ cells, and α‐tubulin was determined to ensure equal loading

It is well known that activation of NF‐κB p65 and its targets involved in the pathogenesis of autoimmune diseases, including IBD.^37^, ^38^ IL‐6, a cytokines critical to the pathogenesis of IBD, was found to dramatically increase in IECs transduced with Lv‐MCT4 compared with controls. To explore whether MCT4‐induced IL‐6 expression could be attributed to upregulation of NF‐κB activity, as shown in Figure 5A‐B, IL‐6 transactivation was significantly increased in HT‐29 cells with MCT4 overexpression, which was markedly reduced in response to NF‐κB depletion, while inhibition of MCT4 by CHC dramatically attenuated IL‐6 transactivation. These phenomena were attributed to the binding of NF‐κB p65 to IL‐6 promoter was drastically increased in response to MCT4 overexpression (Figure 5C). Moreover, silencing of the NF‐κB p65 gene using siRNA resulted in abrogation of IL‐6 expression caused by MCT4 (Figure 5D), indicating that MCT4 has a significant role in NF‐κB‐mediated transactivation of IL‐6.

Our data suggested that MCT4 was essential for NF‐κB‐dependent transcription of IL‐6; the exact mechanism remained unclear. To explore whether MCT4 contributed to IL‐6 expression by promotion of NF‐κB activity, we found that the relative abundance of NF‐κB p65 in nucleus was decreased to 25% of control group by CHC treatment (Figure 5E‐F), and NF‐κB p65 in nucleus was evaluated in colonic epithelial of patient with IBD compared with healthy donors (Figure 5G). In addition, CHC treatment strongly reduced NF‐κB nuclear translocation in CaCO_2_ cells (Figure 5H), whereas there was a significant activation of NF‐κB p65 phosphorylation at Ser536 in HT‐29 and CaCO_2_ cells with MCT4 overexpression (Figure 5I). Conversely, stimulation by CHC in IECs led to a dramatic inhibition of NF‐κB p65 (Ser536) (Figure 5J). Taken together, these results demonstrated that MCT4 mediated IL‐6 expression via activation of NF‐κB pathway.

### 3.6 MCT4 regulated the interaction between CBP and NF‐κB or CREB

**Figure 6:**
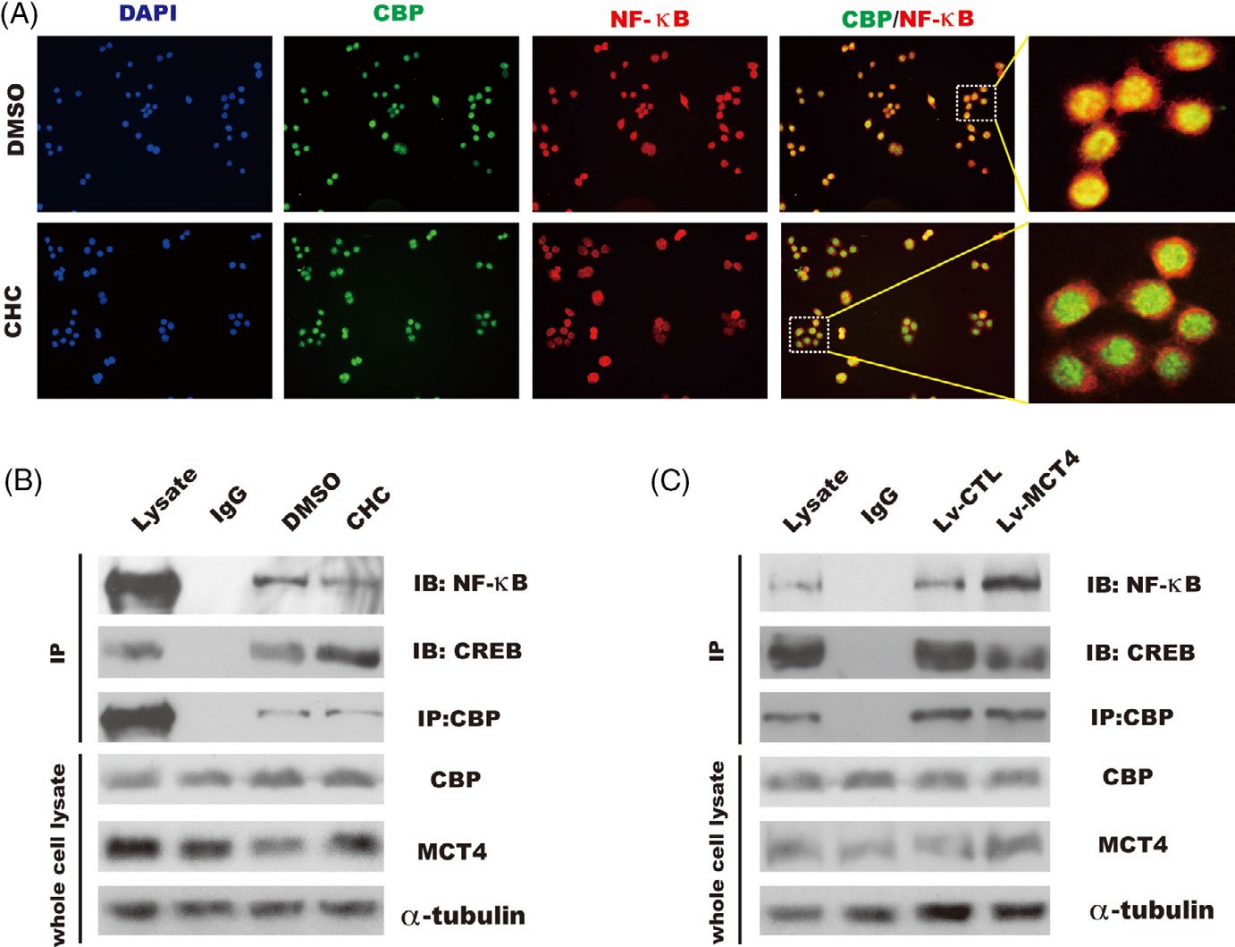
MCT4 modulates the interaction of CBP and NF‐κB or CREB. A, Co‐localization of endogenous CBP and NF‐κB by immunofluorescence performed in HT‐29 cells treated as indicated, respectively, scale bar: 200 μm. B, CaCO_2_ cells were grown to 80% confluence and replaced with medium without serum for 18 h, and then stimulated with DMSO or CHC for 1 h; whole cell lysates were immunoprecipitated (IP) with antibodies against endogenous CBP; and NF‐κB and CREB were detected by immunoblotting. C, Indicated stable CaCO_2_ cells were grown to 80% confluence, whole cell lysates were immunoprecipitated (IP) with antibodies against endogenous CBP, and co‐precipitates with NF‐κB/CREB were detected by immunoblotting

The above results showed that MCT4 contributed to NF‐κB‐mediated inflammatory reaction and decreased CREB‐induced ZO‐1 expression that led to destroy barrier function, a critical step in the transcriptional regulation mediated by NF‐κB or CREB is the interaction of each of these transcription factors with the co‐activator CBP.^39^, ^40^ In order to better understand the driving forces controlling IBD by MCT4, we investigated the potential role of MCT4 in the complex of NF‐κB‐CBP and CREB‐CBP complex in IECs. As shown in Figure 6A, immunofluorescence showed that endogenous NF‐κB was co‐localized with CBP, which was disrupted by CHC stimulation for 1h. Consistent with this result, IP of CBP showed a markedly reduction of NF‐κB‐CBP interaction and a strong increase in CREB‐CBP complex in CaCO_2_ cells with CHC treatment for 1 hour (Figure 6B); in contrary, overexpression of MCT4 significantly disrupted CREB‐CBP interaction and contributed to the formation of NF‐κB‐CBP complex (Figure 6C). These results suggested that the expression level of MCT4 was critical for the interaction of NF‐κB/CREB and CBP.

### 3.7 CHC improved intestinal barrier function and alleviated DSS‐induced colitis in vivo

**Figure 7:**
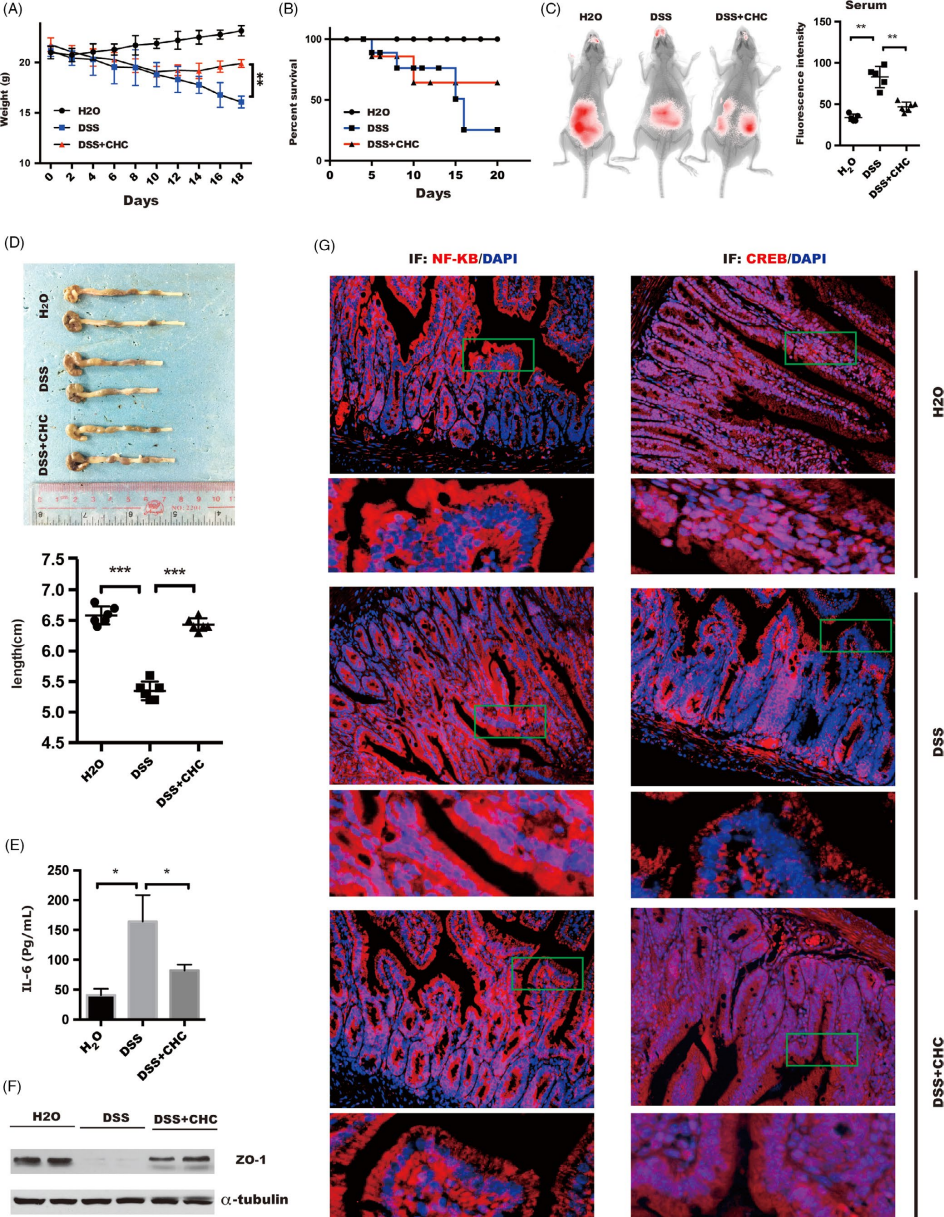
Administration of CHC ameliorates DSS‐induced colitis in mice. Two groups of DSS‐exposed mice (n = 15) were administered intraperitoneally with CHC (10 mmol/L, 100 μL) or vehicle daily starting on day 5‐20 after DSS induction. Another group of mice without receiving water treatment (n = 15) daily on the same schedule as positive controls. (A) The changes in body weight and (B) overall survival were expressed as the percentage of initial survival rate at the start of the experiments as 100%. **P < .01. (C) In vivo detection of orally administered tracer (10 kDa dextran) for 1 hour. Representative in vivo live imaging is shown on the left and quantitation of fluorescent in serum on the right. Data represent the means ± s.d. of three independent experiments and were analysed by one ANOVA, **P < .01. (D) Representative colon length of mice in group treated as indicated (*upper panel*), and statistical significance was performed by one‐way ANOVA, ***P < .001(*lower panel*). (E) The concentration of IL‐6 level in serum was measured in different groups by ELISA assay and analysed by One‐way ANOVA, *P < .05. (F) ZO‐1 expression was analysed via immunoblotting in indicated group. (G) The nuclear location of NF‐κB (*left panel*) or CREB (*right panel*) was detected by immunofluorescence, scale bar: 100 μm

Our study indicated that MCT4 has an important role in the pathogenesis of IBD, and we further assessed the effect of MCT4 inhibitor CHC on intestinal inflammation and intestinal epithelial integrity in vivo. Chronic colitis in C57BL/6 mice induced by 2% DSS in drinking water for 7 days, followed by intraperitoneal administration of CHC or vehicle controls for 14 days. CHC‐treated mice were found to be protected from experimental colitis as predicted (mean bodyweight) and improved overall survival (Figure 7A‐B). Imaging live mice after oral administration of label dextran revealed that CHC treatment resulted in improvement of intestinal barrier integrity: fluorescence in the intestinal lumen was slightly increased in DSS‐induced colitis followed by CHC treatment compared with DSS group (Figure 7C *left panel*). In a confirmatory assay, circulating concentrations of Cy7‐label dextran after gavage in CHC treatment were significantly lower than in DSS group, showing a better barrier function (Figure 7C *right panel*), and a longer colon length compared with the DSS group (Figure 7D). Most importantly, a significant reduction of IL‐6 was observed in DSS + CHC mice compared with that of DSS mice (Figure 7E), which was not only attributed to inhibit NF‐κB nuclear location (Figure 7G left panel), but also enhance CREB nuclear location (Figure 7G right panel) by CHC, leading to increase in ZO‐1 expression (Figure 7F). Taken together, these data suggested that CHC plays a key role in improvement of the intestinal barrier, likely through its effects on ZO‐1 and inflammatory cytokines expression in vivo.

## 4 DISCUSSION

**Figure 8:**
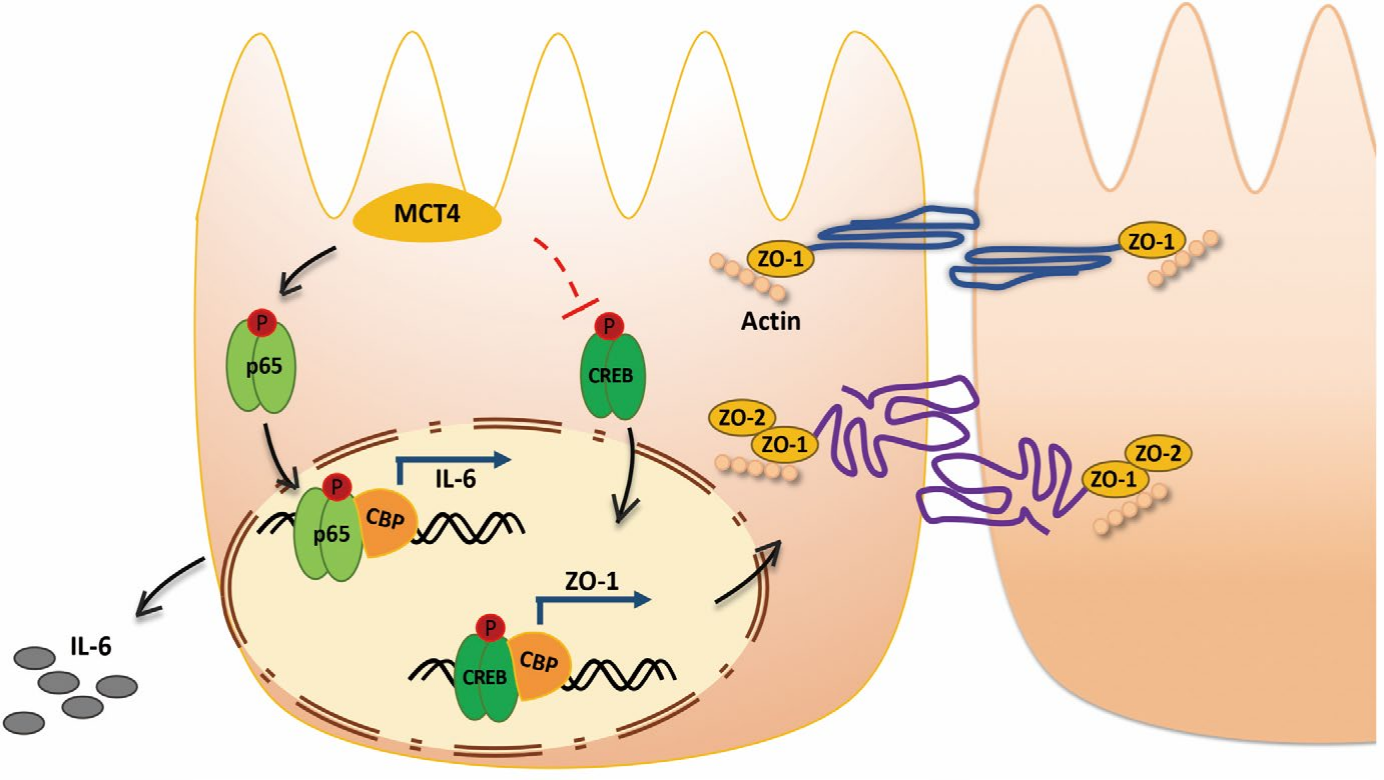
Schematic illustration for the crosstalk of MCT4 and CREB or NF‐κB in regulation of IBD. On one hand, MCT4 primarily mediates phosphorylation of CREB (Ser133) and leads to reduce CREB nuclear translocation and DNA binding to the promoter of ZO‐1. On the other hand, MCT4 leads to NF‐κB p65 phosphorylation and directs NF‐κB p65 nuclear translocation, and DNA binding to IL‐6 promoter. Most importantly, MCT4 contributes to interaction of NF‐κB p65 with CBP, which in turn to disrupt CREB‐CBP complex, depicting the alteration of MCT4 is critical to modulate shift balance between NF‐κB‐CBP and CREB‐CBP complex, and these results implied proper expression of MCT4 is critical to improve IBD

Up to now, no available reports on the function of MCT4 in inflammatory bowel disease. In this study, we further rigorously demonstrated a novel signalling pathway driven by MCT4 in regulating IBD by modulation of shift balance between NF‐κB‐CBP and CREB‐CBP complex. As shown in Figure 8, we, for the first time, reported that the potent pro‐IBD effect of MCT4 stems from its ability to globally upregulate inflammatory factors and attenuate ZO‐1‐mediated intestinal barrier function in IECs through regulating two key transcription factors, NF‐κB and CREB, respectively. Mechanistically, on one hand, MCT4 phosphorylates transcription factor NF‐κB, initiates pro‐inflammatory factor expression and, subsequently, aggravates inflammatory response. On other hand, MCT4 inhibits CREB activity and abolishes ZO‐1 transcription, leading to destroy intestinal barrier function. Alterations of MCT4 expression level are sufficient to induce a switch between CBP‐NF‐κB and CBP‐CREB complex, leading to different biological function in inflammatory bowel disease. Most importantly, MCT4 inhibitor CHC improved intestinal barrier function and alleviated DSS‐induced colitis in vivo*.* Thus, these results provided novel evidence to support an important role of MCT4 as a promising new approach for targeting therapy of patients with IBD.

In the current study, we showed that increased MCT4 expression was positively correlated with IL‐6 levels, while inversely associated with ZO‐1 expression in clinic, and MCT4 overexpression led to markedly increase in intestinal epithelial cell permeability and decrease in TEER value, indicating that MCT4 has a potential role in destroying intestinal barrier function. Moreover, data from qPCR, Western blotting and ELISA demonstrated that MCT4, albeit regulating different target genes, mediated IBD through inhibition of ZO‐1 expression and contribution of IL‐6 expression. In addition, E‐cadherin, JAMA and Claudins were slightly decreased in response to MCT4 overexpression. However, the expression of Occludin and Claudins were unchanged, the redistribution of both is required to be identified.

The NF‐κB signalling pathway is well known to be a central regulator of serval kinds of inflammatory responses.^41^ Activation of NF‐κB induces expression of pro‐inflammatory genes (eg, TNF‐α, IL‐6, IL‐8 and COX‐2).^12^, ^38^ Since MCT4 has a critical role in inflammatory factor expression, investigation of the NF‐κB pathway might help us to further reveal the underlying mechanisms. Stimulation of CHC‐induced suppression of NF‐κB p65 phosphorylation was also found in HT‐29 and CaCO_2_ cells, while ectopic expression of MCT4 significantly increased NF‐κB p65 phosphorylation, leading to NF‐κB nuclear translocation and interaction of CBP with NF‐κB, and contributed to NF‐κB‐DNA binding activity and IL‐6 expression. All of these results demonstrated that MCT4 promoted NF‐κB activation, whether MCT4 enhanced NF‐κB activation in canonical or non‐canonical pathway remains to be determined. Meanwhile, activation of NF‐κB p65 that is translocated to the nucleus where it initiates a complex transcriptional response,^42^, ^43^ which can interact with a transcriptional co‐activator CREB‐binding protein (CBP), leading to displacement of CREB from the same interaction domain on CBP^16^, ^17^ and promotion of the expression of cytokines, typically involved in pro‐inflammatory events, thereby suppressing expression of anti‐inflammatory cytokines activated by CREB‐CBP complex.^19^ In line with this, we found that overexpression of MCT4 inhibited CREB activity and nuclear translocation, resulting in ablation of CREB‐DNA binding activity and formation of CREB‐CBP complex, and decreased ZO‐1 expression.

The function of MCT4 in IBD majorly depends on its expression level, which indirectly modulates the shift balance between the CREB‐CBP interaction and the NF‐κB‐CBP complex. Interestingly, we found the contribution of MCT4 to NF‐KB replaced with CREB, leading to form NF‐κB‐CBP complex, which further not only activated phosphorylation of NF‐κB p65(Ser536) and nucleus translocation, resulting in increase of pro‐inflammatory cytokines expression, such as IL‐6 and TNF‐α, but also attenuated level of CREB(Ser133) activity and nucleus translocation, led to decrease ZO‐1 and anti‐inflammatory cytokines expression. However, the limitation of this study is that changes of MCT4 expression and activity in IBD remain to be addressed, and further work is required to demonstrate how MCT4 regulated CREB/NF‐κB activity, whether in MAPK‐dependent way, although lacking strong evidences to confirm it at present.

In conclusion, normal expression of MCT4 is essential to coordinate IECs epithelial barrier integrity and inflammatory response, and our data have established a novel mechanism for regulation of ZO‐1 and inflammatory factors expression by MCT4. Alterations in MCT4 expression are sufficient to induce a switch between CBP‐NF‐κB and CBP‐CREB complex, leading to different biological function in inflammatory bowel disease. Further studies are warranted to elucidate the MCT4 expression, even MCT4 activity, during development of inflammatory bowel disease under physiological or pathological conditions.

## CONFLICT OF INTEREST

The authors declared that they have no conflict of interests.

## AUTHOR CONTRIBUTIONS

STG, WFX, LLG and SXZ conceived and designed the experiments; HLW, MSL, KJZ, YH, MWC, YC, GHC, YWX, ZHX, RTL, SYL and YDW performed experiments and analysed data; WFX and LLG wrote the manuscript; WFX and STG revised manuscript. Specifically, MWC, ZHX and KJZ performed most work in revised stage. All authors read and approved the final revised manuscript.

## ETHICAL APPROVAL

Human study: Based on the declaration of Helsinki as reflected in a prior approval by the institution's human research committee, this study was conducted in a cohort of 54 patients with inflammatory bowel disease (IBD) and 16 healthy control in Guangzhou Women and Children's Medical Center from 2016 to 2018 approved by Guangzhou Women and Children's Medical Center animal care and use committee. Written informed consent was given by the caregiver of the child for his clinical records used, which are not publicly available since the database is currently not anonymous and contains all patient's name; however, it could be available upon request. Animal study: All animal experiments were approved by Southern Medical University animal committee and performed at Southern Medical University.

## Data Availability

The data that support the findings of this study are available from the corresponding author upon reasonable request.
