# Supplementary figures and images for: Inhibition of CREB‐mediated ZO‐1 and activation of NF‐κB‐induced IL‐6 by colonic epithelial MCT4 destroys intestinal barrier function
Source: Cell Prolif. 2019 Aug 16;52(6):e12673. doi: 10.1111/cpr.12673 (PMC6869122; doi:10.1111/cpr.12673)

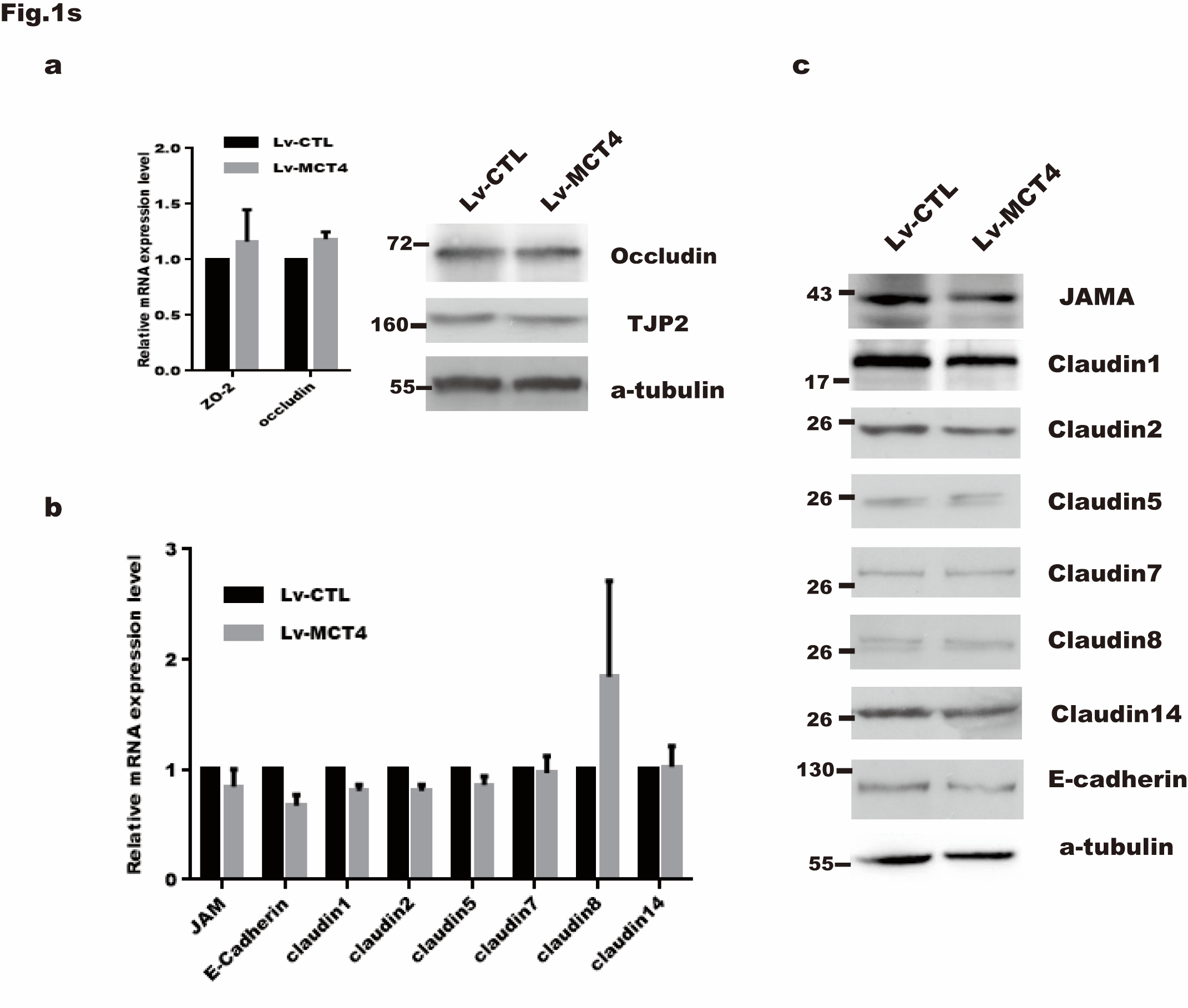

Supplement: Supplementary file 1 [file CPR-52-e12673-s001.tiff]
